# An exploration of the non‐iterative time trade‐off method to value health states

**DOI:** 10.1002/hec.3773

**Published:** 2018-05-17

**Authors:** Yan Feng, Arne Risa Hole, Milad Karimi, Aki Tsuchiya, Ben van Hout

**Affiliations:** ^1^ Office of Health Economics London UK; ^2^ Department of Economics University of Sheffield Sheffield UK; ^3^ Erasmus School of Health Policy & Management Erasmus Universiteit Rotterdam Rotterdam The Netherlands; ^4^ School of Health and Related Research University of Sheffield Sheffield UK

**Keywords:** exhaustion of lead time, general public health state preferences, iteration bias, online survey, states worse than dead

## Abstract

Time Trade‐Off (TTO) usually relies on “iteration,” which is susceptible to bias. Discrete Choice Experiment with duration (or DCE_TTO_) is free of such bias, but respondents find this cognitively more challenging. This paper explores non‐iterative TTO with or without lead time: NI(LT)TTO. In NI(LT)TTO, respondents see a series of independent pairwise choices without iteration (similar to DCE_TTO_), but one of the two scenarios always involves full health for a shorter duration (similar to TTO). We compare three different “types” of NI(LT)TTO relative to DCE_TTO_. Each type is presented in two “modes”: (a) verbally tabulated (as in a DCE) and (b) with visual aids (as in a TTO). The study has 8 survey variants, each with 12 experimental choice tasks and a 13th task with a logically determined answer. Data on the 12 experimental choices from an online survey of 6,618 respondents are modelled, by variant, using conditional logistic regressions. The results indicate that NI(LT)TTO is feasible, but some relatively mild states appear to have implausibly low predicted values, and the range of predicted values is much narrower than in DCE_TTO_. The presentation of NI(LT)TTO tasks needs further improvement.

## 1 INTRODUCTION

Preference‐based health state classification systems require health state valuations. Conventional Time Trade‐Off (TTO) protocols such as those used to value the EQ‐5D (Dolan, 1997) have two main methodological challenges. First, it relies on “iteration”—a series of pairwise choice tasks deals with a given hypothetical health profile (i.e., a health state for a specified duration), where the tasks are identical except for (shorter) duration in full health and are iterated in a systematic (and predictable) ordering until indifference is achieved for the health profile. The literature on willingness to pay would suggest that such procedures are susceptible to bias arising from the iteration process (Ternent & Tsuchiya, 2013). Discrete Choice Experiment (DCE) is a method that does not involve iteration because each task is independent and is therefore free of iteration bias. DCE with duration as an attribute (DCE_TTO_) can generate health state values anchored at 1 for full health and 0 for being dead at the aggregate level (Bansback, Brazier, Tsuchiya, & Anis, 2012). However, a disadvantage of DCE_TTO_ is the cognitive burden: each pairwise choice task in a DCE_TTO_ of EQ‐5D‐5L comprises 12 pieces of information (the five dimensions of health and duration for one scenario, and the same for the other scenario), and all of these can change from one task to another. There is evidence that respondents find this cognitively more challenging than a conventional TTO, where only one piece of information (duration in full health) changes from one task to another for a given health profile (Mulhern et al., 2014).

The second methodological challenge for conventional TTO is that it has separate protocols depending on whether the state is better or worse than being dead, and data for the latter are typically subjected to arbitrary transformation (Patrick, Starks, Cain, Uhlmann, & Pearlman, 1994). DCE_TTO_ does not require a separate protocol or transformation but has its challenges (see above). The addition of lead time has been suggested as an alternative way of valuing both kinds of states using a uniform protocol (Devlin, Tsuchiya, Buckingham, & Tilling, 2011). This, in effect, allows the shorter duration in full health to take negative values and requires no arbitrary transformation of data. However, respondents may “exhaust” lead time (Devlin et al., 2011; Devlin et al., 2013), where a given lead time is not long enough to accommodate their preference for a state worse than dead. For example, if a respondent prefers to die immediately rather than to live in full health for 10 years (lead time) followed by 10 years in a severe state, then lead time TTO cannot determine an indifference point, only that the health state value is strictly lower than −1.

One possible next step is a non‐iterative variant of TTO. Non‐iterative TTO (NITTO) *without* lead time is a cross between iterative TTO and DCE_TTO_. The respondent is faced with a series of independent pairwise choices between health profiles, without iteration (so it is like a DCE_TTO_), but one of the two scenarios always involves full health for a shorter duration than the other scenario (so it is like a TTO). The motivations are to avoid the iteration bias of TTO and to reduce some of the cognitive burden associated with DCE_TTO_. However, it is not clear if NITTO can predict values for states worse than dead with high precision—it will not have direct observations in the negative range and will need to extrapolate negative values based on ordinal preferences observed in the positive range. A possible adaptation is NITTO *with* lead time (NILTTTO), which simply adds lead time to NITTO scenarios. The obvious disadvantage of this is the added complexity, while the advantage is that, because the preferences elicited are ordinal, the analysis of NILTTTO data is not hindered by the exhaustion of lead time.

Table 1 summarises the main advantages and disadvantages of these different methods. The abbreviation “NI(LT)TTO” is used throughout this paper to mean non‐iterative TTO with or without lead time. An NI(LT)TTO exercise was first explored using the three‐level EQ‐5D in Mulhern et al. (2014), which referred to it as “binary choice TTO,” but we will call it “non‐iterative TTO,” because most TTO exercises (including those that are iterative) are based on a series of binary choice tasks. Mulhern et al. (2014) demonstrated that respondents can deal with non‐iterative TTO tasks. However, not much else is known about this innovative method.

**Table 1:** Summary of TTO, DCETTO, and NI(LT)TTO

	Advantages	Disadvantages
TTO	One value per respondent per state	Iteration bias
	Effective visual aid	Separate task for <0
	Relatively simple tasks	Arbitrary transformation for <0
LT‐TTO	One value per respondent per state	Iteration bias
	Same task for <0	Lead time looks complicated
	Effective visual aid	Exhaustion of lead time
DCE_TTO_ (Type 0)	No iteration	No cardinal value per respondent
		A lot of information to process
		Lots of change from task to task
	Same task for <0	
NITTO (Type 1)	No iteration	No cardinal value per respondent
	No separate task for <0	
	Relatively simple tasks	Need to extrapolate <0
NILTTTO (Types 2, 3)	Same task for <0	No cardinal value per respondent
	No exhaustion of lead time	Lead time looks complicated

The aim of this paper is to build on earlier research to explore the effects of the following:

- respondents with various data quality concerns;
- different “types” of NI(LT)TTO designs;
- different “modes” of presenting pairwise choice tasks;
- learning and fatigue effects over 12 choice tasks; and
- heterogeneity in respondent preferences.

## 2 METHODS

The survey consists of four types of experimental tasks (0, 1, 2, and 3), presented in two different modes of presentation (a and b), resulting in eight survey variants. Example screenshots from variants 0a (viz. Type 0 with Mode a), 1b, 2a, and 3b are appended.

### 2.1 The four types of experimental tasks

All the choice tasks used in this study use EQ‐5D‐5L (Herdman, Gudex, & Lloyd, 2011) to describe health states. Each choice task involves two scenarios consisting of “you” living in a hypothetical EQ‐5D‐5L state or in “full health” for a specified duration followed by death, where respondents are asked which scenario they think is better. No ties are allowed. Of the four types, the first (DCE_TTO_) is included as a baseline against which to compare the NI(LT)TTO types against. The second (NITTO) is the natural hybrid of DCE_TTO_ and iterative TTO. The third and fourth add lead time to this (NILTTTO) but are designed using different approaches.

Type 0 The baseline type used in the study is a DCE_TTO_ that replicates the design used in an earlier study (“Type Ia” from Mulhern, Bansback, Hole, & Tsuchiya, 2017). This consists of 120 scenario pairs generated using Ngene (Choice Metrics, 2012) and has six levels of duration (6 months, 1, 2, 4, 7, and 10 years). (The number of scenario pairs, 120, is sufficient to estimate a model with categorical dummies representing the EQ‐5D‐5L descriptive system and continuous duration; interactions between the descriptive system and duration; quadratic duration; and interactions between the descriptive system and quadratic duration—for details, see Mulhern et al., 2017). Prior values of zero are used for all parameters, and no adjustment is made for so‐called “implausible” states. A DCE_TTO_ task can be represented as a choice between two scenarios, or health profiles, A and B, where the levels of utility *u* associated with each health profile, made up of state *x* for duration *t*, are given by *u*_*A*_ = *βt*_*A*_ + *λx*_*A*_*t*_*A*_ + *ε*_*A*_, and similarly, *u*_*B*_ = *βt*_*B*_ + *λx*_*B*_*t*_*B*_ + *ε*_*B*_, where *β* represents the utility of living in full health for 1 year (expected to be positive); and *λ* represents the (dis)utility associated with living with health problems *x* for 1 year (expected to be negative). The associated value of health state *x* (*v*_*x*_) is given by

$$v_{x}=1+\frac{\hat{\lambda }}{\hat{\beta }}\;x.$$

This formula applies to all four types. Under Type 0, negative values are interpolated from within the data, when the combined disutility of an EQ‐5D‐5L state cancels out the utility of full health. For further details, see Bansback et al. (2012).

Type 1 This is a non‐iterative TTO with no lead time (NITTO). One scenario (A) is to live in an EQ‐5D‐5L state for 10 years, whereas the other (B) is to live in “full health” for one of six shorter durations (6 months, 1, 2, 4, 6, and 9 years). This can be represented as $u_{A}=\beta \bar{T}+\lambda x_{A}\bar{T}+\varepsilon_{A}$ and *u*_*B*_ = *βt*_*B*_ + *ε*_*B*_, where $\bar{T\;}>t$. The choice tasks correspond to TTO for states better than being dead, and therefore negative values are extrapolated from observations in the positive range. Ngene is used to generate 120 scenario pairs using prior values of zero constrained to be full health.
Type 2 This is a non‐iterative TTO with lead time (NILTTTO). It is similar to Type 1, but the six levels of duration used in scenario A includes a “negative” level of duration (−3, 0, 3, 5, 7, and 9 years). The design is identical to that of Type 1, except for the labels attached to the different levels of duration—the experimental design uses zero prior values, so is unaffected. In order to operationalise the negative durations, a 4‐year lead time is used: in the actual choice tasks, scenario A is 4 years in “full health” followed by 10 years in the EQ‐5D‐5L state, whereas scenario B is 1, 4, 7, 9, 11, or 13 years in “full health.” In the analysis, the lead time is subtracted so that $\bar{T}$ is 10 and *t* ranges from −3 to 9.
Type 3 This is another NILTTTO (NILTTTO‐II), but instead of an experimental design to select pairs of health *scenarios* in a single step, a two‐stage design is used. This is an innovative and promising approach to design DCE_TTO_ (Mulhern et al., 2017), and the present study tests if this is also viable for NILTTO. In the first stage, 120 pairs of EQ‐5D‐5L *states* (with no durations) combined with full health are generated, assuming *u*_*A*_ = *λx*_*A*_ + *ε*_*A*_ and *u*_*B*_ = 0. In the second stage, each state *x* in scenario A is matched with one of the six duration levels *t* for scenario B (−3, 0, 3, 5, 7, and 9 years) that achieves an expected split of respondents between the two scenarios of 70% versus 30%, which is chosen to be within the range of optimal choice probabilities for DCEs derived by Kanninen (2002). The results of Mulhern et al. (2014) were used as priors for this second stage. As with Type 2, the lead time in “full health” is added for the presentation to respondents but removed for the analysis.

### 2.2 The modes of presentation

NI(LT)TTO is a cross between iterative (LT)TTO and DCE_TTO_ and can be presented as either of these. Typically, (LT)TTO are presented using visual aids or TTO boards, whereas DCE exercises are presented using tabular format. Thus, each of the four types above is presented in two different modes.

Mode (a) a tabulated presentation taken from Bansback et al. (2012), Mulhern et al. (2014), and Mulhern et al. (2017); and
Mode (b) a visual aid similar to TTO boards used in Gudex (1994), Dolan (1997), and Devlin et al. (2013).

### 2.3 Survey design, recruitment, and the sample

Within each type, the 120 choice sets are blocked randomly into 10 blocks of 12 tasks using Stata (Stata Corp); this procedure is repeated 10 times, and the blocking variable with the lowest association with the design attributes is chosen as the final blocking variable. Each respondent within a given variant is randomly allocated 1 of the 10 blocks.

Data were collected through an online survey using a commercial internet panel (Survey Sampling International). Age and sex quotas were set for each of the eight survey variants corresponding roughly to the UK general population. Target sample size was 600 for Type 0 (DCE_TTO_) and 900 for the other types. Panel members were invited by e‐mail to take part in one of the survey variants. Part 1 of the survey consisted of background questions including age, sex, education, own EQ‐5D‐5L, and life satisfaction. Part 2 was for the 12 choice tasks. In addition, there was a 13th task with a logically determined answer, which was the same across all variants (A: mild state for 10 years; vs. B: full health for 10 years) but presented in the relevant format. Part 3 of the survey asked additional questions including assessment of the choice tasks. The survey was hosted by epiGenesys, a University of Sheffield spin off company.

### 2.4 Analyses of quantitative data

The choice data by respondent *i* for scenario *j* are modelled using conditional logit regressions:

$$u_{\mathrm{ij}}=\beta t_{\mathrm{ij}}+\lambda \;x_{\mathrm{ij}}t_{\mathrm{ij}}+\varepsilon_{\mathrm{ij}}.$$

Of particular interest are the sign and significance of the *β* and the *λ* coefficients (*β* is expected to be positive and *λ* is expected to be negative); the relative ordering and significance of the *λ* coefficients within dimensions (e.g., whether the Level 4 coefficient for self‐care is statistically significantly worse than the Level 3 coefficient of the same dimension); predicted health state values for select states (22222, 33333, 44444, and 55555); and the gap between the predicted values for states 22222 and 55555.

There are two further quantitative analyses.

First, learning and fatigue effects are explored, through modelling the early tasks (Tasks 1–4), middle tasks (Tasks 5–8), and the late tasks (Tasks 9–12) separately, for each variant.

Second, heterogeneity in respondent preferences is examined through latent class analysis, by variant, using the *lclogit* command in Stata (Pacifico & Yoo, 2013). This analysis assumes that respondents can be divided into subgroups (or classes) depending on their preferences. In the estimation process, a separate set of coefficients is estimated for each class (Greene & Hensher, 2003; Hole, 2008), reflecting that preferences are allowed to vary across, but not within, classes.

Stata Versions 13 and 14 are used for all analyses.

### 2.5 Analysis of free text comments

Before the end of the survey, participants were given a chance to leave a comment in a textbox field. The comments are reviewed with the aim to develop overarching themes and to compare these themes across variants. They are categorised into themes in several steps. Each comment of the first variant (0a) is assigned an initial theme and an initial index was developed. This index is applied to the next variants. A new one is created if an appropriate theme is not available for a comment. Comments could be classified under multiple themes. The themes and the number of times each theme was mentioned are then reviewed to see if differences existed per variant.

## 3 RESULTS

### 3.1 Response rate and demographics

There are no large discrepancies in respondent numbers and rates across variants in completion rates, although respondents allocated to Type 0 (DCE_TTO_) take longer than the rest (see Table A1). In general, respondents' background characteristics are similar across the eight variants (Table A2).

### 3.2 Descriptive statistics of the choice tasks by variant

The median time taken for individual choice tasks, by variant, is reported in Table 2. Respondents spend considerably more time in the first task than the remaining 12 tasks. Task 13 (the logical consistency test) does not seem to take less (or more) time than the preceding tasks. Looking at the averages of the individual median time taken for Tasks 2–12, Type 0 (DCE_TTO_) takes the most and Type 1 (NITTO) takes the least time. There is little difference by mode amongst these types. Across Types 2 and 3 (NILTTTO and NILTTTO‐II), the effect of Mode is larger than the effect of type and the tabulated variants (Mode a) take less time than the visual aid variants (Mode b).

**Table 2:** Descriptive statistics of the choice tasks by variant

		0a	0b	1a	1b	2a	2b	3a	3b
Number of respondents		618	593	901	900	901	902	903	900
Time taken in seconds	Task 1^a^	87	103	78	96	76	109	72	120
	Task 2^a^	21	19	16	14	13	18	13	18
	Task 3^a^	20	20	14	14	12	16	12	16
	Task 4^a^	19	19	13	12	12	14	12	16
	Task 5^a^	17	18	12	13	11	16	11	15
	Task 6^a^	18	19	13	13	11	15	10	14
	Tasks 7–11 not shown (available on request)								
	Task 12^a^	15	16	11	11	10	12	10	13
	Task 13^a^	15	14	13	11	10	12	10	14
Average time for Tasks 2–12^b^		17.7	18.1	12.5	12.1	10.8	14.3	10.7	14.4
Preference for B over A for	Tasks 1–12 (%)^c^	48.8	51.5	65.6	68.9	66.8	63.7	62.4	62.8
	Tasks 1–4 (%)^c^	43.9	48.0	65.7	71.4	69.0	68.5	62.5	60.2
	Tasks 5–8 (%)^c^	50.0	51.7	65.2	69.0	66.3	59.1	61.9	63.9
	Tasks 9–12 (%)^c^	52.6	54.7	65.9	66.2	65.1	63.6	62.7	64.4
Always choose B over A for Tasks 1–12 (%)^d^		0.49	0.34	14.65	23.33	13.43	6.76	17.50	13.67
Always choose A over B for Tasks 1–12 (%)^d^		0.32	0.84	2.77	1.78	2.33	0.55	6.87	2.89
Logically correct choice (B) in Task 13 (%)		92.4	92.9	91.2	93.2	91.1	92.8	91.1	90.4
Presentation clear (%)^e^		97.4	96.6	97.3	97.3	97.1	94.6	96.5	90.8
Difficult to imagine states (%)^f^		26.7	26.8	17.4	21.7	18.0	22.0	17.5	23.7
Confident about choice (%)^g^		90.0	90.2	92.5	93.3	92.7	92.5	92.7	89.2
Abstract and unrealistic (%)^h^		41.8	44.0	36.3	41.2	31.3	37.7	35.7	39.4
Interesting exercise (%)^i^		94.0	93.3	92.5	91.1	92.8	91.0	92.1	88.9
Did not know which to choose (%)^j^		71.0	67.8	46.6	52.8	45.8	53.9	48.5	55.7
Able to answer a few more (%)^k^		85.0	83.0	86.0	84.8	85.9	82.5	85.8	78.7

a Median time taken in seconds.

b Average of the median time taken in seconds for Tasks 2–12.

c Percentage of observations across respondents that choose B (shorter survival in full health) over A (longer survival in suboptimal health).

d Percentage of respondents that always choose B over A; or A over B.

e Percentage agreeing to statement: “The presentation of the tasks was very clear.”

f Percentage agreeing to statement: “I had difficulty imagining the health states.”

g Percentage agreeing to statement: “I am confident about my choices.”

h Percentage agreeing to statement: “Some of the health states seemed very abstract and unrealistic.”

i Percentage agreeing to statement: “The exercise was interesting.”

j Percentage agreeing to statement: “Sometimes I really didn't know which one to choose.”

k Percentage agreeing to statement: “I would be able to do a few more of these questions.”

In terms of the distribution of preferences across the two scenarios, A and B, the data in Type 0 (DCE_TTO_) are balanced evenly across the two scenarios, and few (<1%) respondents choose one or the other scenario throughout. For the remaining the six variants, there is a stronger preference for scenario B (shorter survival in full health) over A (longer survival in suboptimal health), and this is observed throughout the 12 tasks. There are no clear associations between these patterns and respondent background characteristics. 1Results available from authors on request. Over 90% of the respondents “pass” the logical consistency test by correctly choosing scenario B, with similar rates across all variants. Table 2 also reports the respondents' assessment of the choice tasks: respondents who were allocated to DCE_TTO_ tasks (0a and 0b) found the tasks more difficult than the others; and amongst the NI(LT)TTO variants, those allocated to the visual Mode (b) report more difficulty than those allocated to the tabulated Mode (a). Nevertheless, there is little variation in the consistently high proportion of respondents who felt they could answer more tasks.

### 3.3 The choice model results

The conditional logit regressions by variant are summarised in Table 3 (see Table A3 for full results). The coefficient for duration is significant in all models, and with the expected sign (positive). Variants 0a, 0b, and 1a do not perform well in terms of number of significant coefficients (variant 0b shows a coefficient with the unexpected sign for Level 2 mobility interacted with duration, or “MO2xD”). Variants 0a and 3a performed the best in terms of the number of coefficients in the expected ordering, followed by variants 2a and then 0b, 1a, and 1b. Statistical significance of each interaction term relative to the adjacent level before (within the same dimension) is also reported. Two of those differences are in the wrong order and are statistically significant at the 5% level (asterisk with a dash): between AD5xD and AD4xD (1b); and between UA5xD and UA4xD (2a). Across the dimensions, most variants result in the largest Level 5 decrement in PD and AD; and the smallest Level 5 decrement in dimensions SC, UA, or MO. In this respect, variants 1b and 3b appear to have unusual ordering of dimensions.

**Table 3:** Summary of the model performance by variant (full sample)

		0a	0b	1a	1b	2a	2b	3a	3b
Duration not *p* < .05		0	0	0	0	0	0	0	0
Interactions not *p* < .05 (*n* out of 20)		4	3	3	0	0	0	0	0
Wrong sign (*n* out of 20)		0	1	0	0	0	0	0	0
Not ordered (*n* out of 20)		1	3	3	3	2	4	1	6
Gap between adjacent levels^a^	MO2xD–MO1xD = 0			***	***	***	***	***	***
	MO3xD–MO2xD = 0								
	MO4xD–MO3xD = 0	**	***	***	**	***		***	***
	MO5xD–MO4xD = 0	*			**			*	
	SC2xD–SC1xD = 0		**		***	**	***	***	***
	SC3xD–SC2xD = 0	**				**		**	
	SC4xD–SC3xD = 0	***	***	***	***	***	*	***	***
	SC5xD–SC4xD = 0	*	***		*				
	UA2xD–UA1xD = 0		**	**	***	***	***	***	***
	UA3xD–UA2xD = 0	*						**	
	UA4xD–UA3xD = 0		***	***	***	***		**	***
	UA5xD–UA4xD = 0	***	**			*'			
	PD2xD–PD1xD = 0	***		***	***	***	***	***	***
	PD3xD–PD2xD = 0	*						***	
	PD4xD–PD3xD = 0	***	***	***	***	***	***	***	***
	PD5xD–PD4xD = 0	**	***	**	**				
	AD2xD–AD1xD = 0	**	***	***	***	***	***	***	***
	AD3xD–AD2xD = 0							***	
	AD4xD–AD3xD = 0	***	***	***	***	***	***	***	***
	AD5xD–AD4xD = 0		***		*'				
Two worst attributes at L5^b^		PD/AD	AD/PD	PD/AD	PD/SC	PD/AD	PD/AD	PD/SC	AD/M
Two least bad attributes at L5^c^		SC/M	M/UA	UA/SC	UA/AD	UA/M	UA/SC	UA/M	PD/UA
Predicted values ( $\hat{v}$)	$\hat{v}$(22222)– $\hat{v}$(55555)	1.37	1.44	1.35	1.00	0.97	0.48	1.13	0.67
	95% CI [ $\hat{v}$(22222)– $\hat{v}$(55555)]^d^	[1.12, 1.61]	[1.15, 1.72]	[1.14, 1.56]	[0.81, 1.18]	[0.76, 1.18]	[0.29, 0.68]	[0.99, 1.26]	[0.52, 0.82]

*Note*. Asterisks with a dash indicate a significant difference in the unexpected direction.

a Null hypothesis: coefficients of adjacent levels are no different.

b Worst/second worst dimensions amongst the Level 5 coefficients.

c Least/second least bad dimensions amongst the Level 5 coefficients.

d Bootstrapped 1,000 times.

* *p* < .05;

** *p* < .01;

*** *p* < .001

**Figure 1:**
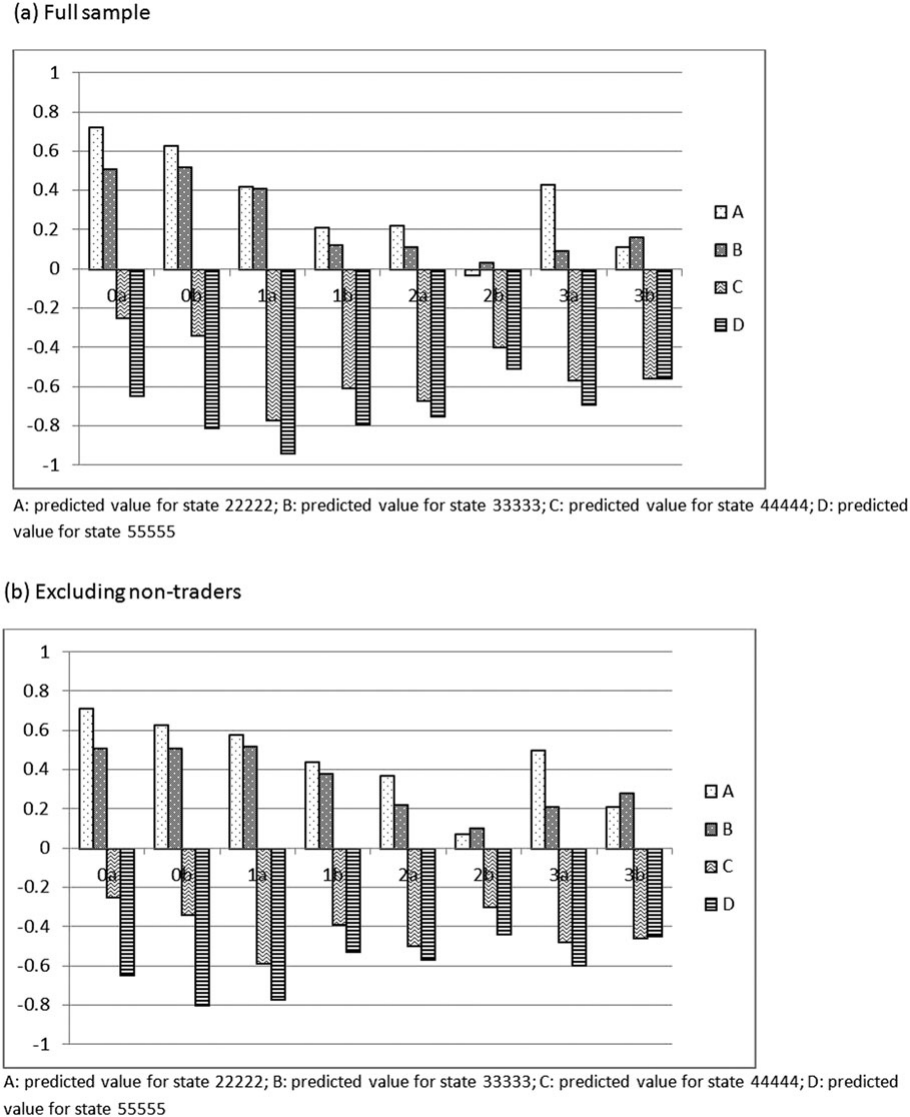
Predicted health state value of four states, by variant (a) Full sample (b) Excluding non‐traders

Predicted values ( $\hat{v}$) for a select number of EQ‐5D‐5L states are illustrated in Figure 1a using the full sample. Predicted values for state 22222 is labelled A, state 33333 is B, state 44444 is C, and state 55555 is D, and the bars are grouped by variant. With one exception, states 22222 and 33333 have positive predicted health state values, whereas states 44444 and 55555 are negative. Values for 22222 and 33333 by DCE_TTO_ are larger than by NI(LT)TTO, but there is no clear pattern for 44444 and 55555. The values for state 22222 from all variants are lower than that from previous studies (e.g., with DCE_TTO_, 0.72 for 0a, 0.63 for 0b, whereas 0.88 in Mulhern et al., 2017). In particular, for variant 2b, the predicted value for state 22222 is worse than being dead (−0.03). Variants 2b and 3b reported higher predicted values for state 33333 than 22222, which is due to the number of coefficients with the unexpected ordering between Levels 2 and 3 in these two variants. The differences between the predicted values for states 22222 and 55555, reported in Table 3 with 95% confidence intervals, are significantly lower than 1 in the two visual NILTTTO variants (2b and 3b). The DCE_TTO_ in Mulhern et al. (2017) has a corresponding value of 1.32.

### 3.4 Further quantitative analyses

The number of respondents who “fail” the logical consistency test is very small, and removing them from the analysis has negligible impact on the regression results. 2Outputs not reported here—available from authors on request. The number of respondents who choose either scenario A or B throughout is not negligible, and their prevalence is not random across variants. However, (with the exception of 1a, resulting in one more non‐significant coefficient) the effect of removing such respondents is not large (see Table A4). Figure 1b illustrates the predicted values for select states without the “non‐traders.” While Type 0 is not affected by the inclusion or exclusion of such respondents, the predicted values are consistently higher (by 0.14 on average) across the NI(LT)TTO variants and the predicted values become more similar across variants.

**Figure 2:**
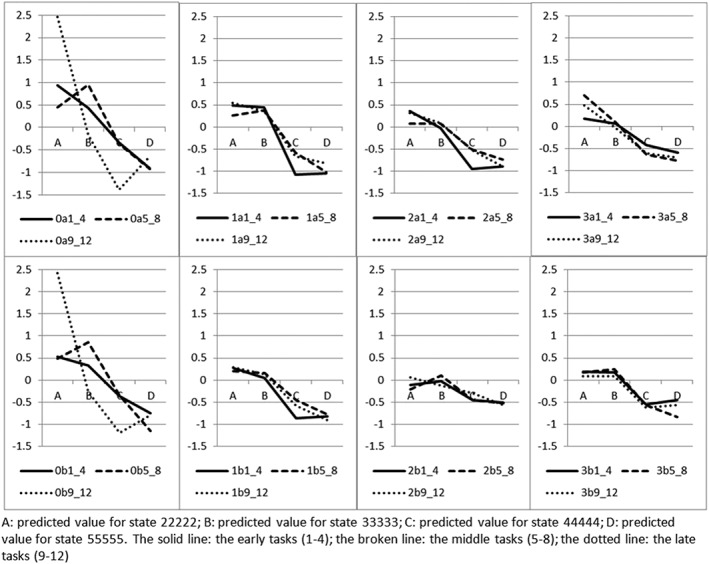
Predicted health state value of four states, by variant, by stages of the tasks

Figure 2 summarises the effects of modelling the data by breaking up the 12 tasks into early tasks (Tasks 1–4), middle tasks (Tasks 5–8), and late tasks (Tasks 9–12) by variant. 3Full results available from authors on request. The solid lines are for the first four tasks (“1_4”); the broken lines are for the next four tasks (“5_8”); and the dotted lines are for the last four tasks (“9_12”). The three lines, logically, should be non‐increasing, which is clearly not the case for middle (broken) and late (dotted) tasks in Type 0. While preferences appear to be stable across the 12 NI(LT)TTO tasks, 12 DCE_TTO_ tasks is possibly too demanding.

**Figure 3:**
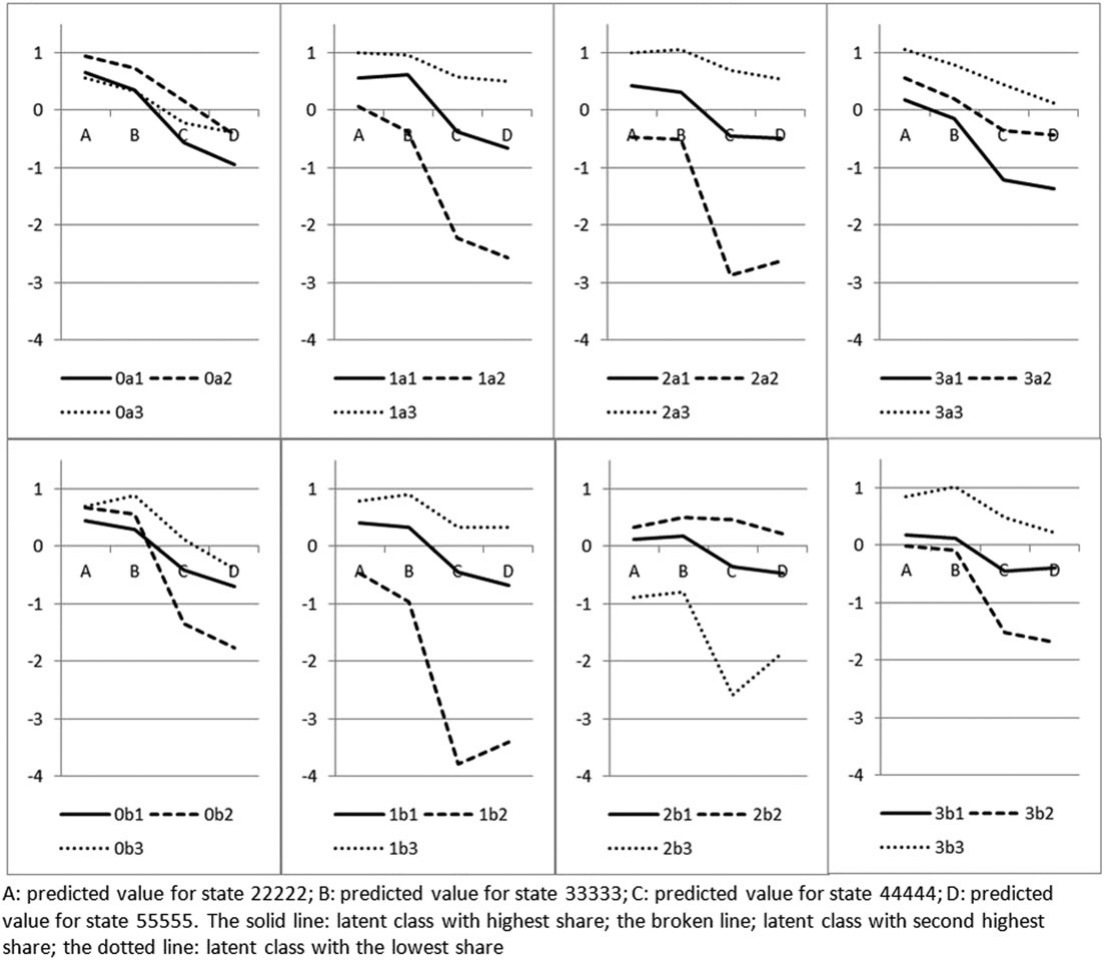
Predicted health state value of four states, by variant, by latent class

Figure 3 summarises the heterogeneity in preferences across respondents. Three latent classes of preferences are assumed for each variant, and the predicted values for key states are plotted. The class with the highest share is labelled Class 1 (solid lines; these on average have around a 45% share); the class with the second highest share is labelled Class 2 (broken lines; 33%); the least prevalent is Class 3 (dotted lines; 21%). 4Full results available from authors on request. Again, all the lines should be nonincreasing, which is not the case for Class 1 (solid) variants 1a, 3b; Class 2 (broken) 1b, 2a, 2b; and Class 3 (dotted) 0b, 1b, 2b, 3b. The graphs show that the data in variants 0a are relatively homogeneous (the lines are close together), whereas the data in Types 1 and 2 are much more heterogeneous. Except for variant 3a, predicted values for the most prevalent class (1, broken) are positive for states 22222 and 33333, whereas negative for 44444 and 55555. Some classes (1a3; 1b3; 2a3; 2b2; 3a3; 3b3) have four positive predicted values, which implies the absence of any state worse than dead (which is possible); while other classes (1b2, 2a2, 2b3, 3b2) have four negative predicted values, which has limited face validity.

### 3.5 Qualitative analysis of free text comments

Overall, 1,530 comments were coded (including multiple categories per comment) into 29 themes. 5Full results available form authors on request. Table 4 summarises the 10 most frequent themes, within each variant. Example quotes for each theme are presented in Table 5. These themes cover 86–96% of the comments, depending on the variant. Across all variants, the most frequent are positive comments (26% for 3b; to 40% for 2a). Explanation of how a respondent made their choices was another frequent theme. These often referred to not wanting to be a burden on others. Comments for the NI(LT)TTO variants focused often on the trade‐off between quality of life and survival, rather than between health dimensions. For example

I was expecting to have to make more difficult choices ‐ e.g. choosing between anxiety/depression and pain. In the event I always chose the full health option (even if only 1 year was offered) because I believe full health is priceless (variant 3b).

**Table 4:** The distribution of the overall top 10 themes within the variants

	Positive (%)	Explaining choices (%)	Made me think (%)	Other (%)	Difficult (%)	Needs more information about life with health state (%)	Difficult to imagine (%)	Uncomfortable (%)	Unrealistic (%)	Relates to previous experience (%)	Sum of top 10 (%)
0a	39	9	5	6	9	2	5	9	4	2	91
0b	33	10	11	6	14	2	8	7	3	1	96
1a	36	14	10	5	3	4	5	7	2	1	89
1b	30	14	8	8	6	15	3	3	6	1	94
2a	40	11	9	7	2	7	1	2	6	5	90
2b	31	10	10	8	4	4	5	3	6	4	86
3a	27	12	13	12	4	5	4	4	2	5	87
3b	26	11	11	9	7	3	8	3	5	3	86
Overall	33	12	10	8	6	5	5	4	4	3	90

*Note*. Percentages represent the share of a given theme within all the comments given in the variant. The columns are ordered by the overall row, pooling across all variants.

**Table 5:** Example quotes for the Top 10 themes

Theme	Example quote
Positive	“it was a very good survey” “the survey was easy to understand well laided out a pleasure to complete” “FAB SURVEY”
Explaining choices	“my choices were based on the fact that, i hate pain and also hate being a burden to anyone including family” “I tended to opt for the choice which gave me the best healthy years. When I chose the alternative one I was intending to do away with myself before I became too dependent on carers.” “It was an interesting survey. Not a situation you normally think about unless you are in it. I think overall my choices were right. I would rather live a shorter life in full health than a longer one in pain and being dependant on another person for everyday task like washing and dressing.”
Made me think	“Made you think about your own life” “Interesting to try to decide whether a short, healthy life is preferable to a longer, but possibly more demanding, life. A really good survey, has given me much to think about. Well designed and set out.” “Very interesting and thought provoking.”
Other	“just be your‐self and manage your time and food”
Difficult	“Anxiety and depression are difficult to compare to pain.”
Needs more information about life with health state	“There is no info about where you would be living or if you had enough money. You can put up with a lot if you are home with someone you love.”
Difficult to imagine	“Difficult to imagine what ‘severe’ depression or pain would feel like.”
Uncomfortable	“I found this entire survey very uncomfortable”
Unrealistic	“Some of the scenarios had totally unrealisitic combinations, e.g. no difficulty walking around but unable to wash and dress yourself. If the scenario does not make logical sense, it is hard bordering on imposible to make a judgement about it.”
Relates to previous experience	“a very interesting survey that was relevant to me after receiving an Industrial Accident of crush spinal injuries”

Between 2% and 14% of comments expressed difficulty of the task, with the DCE_TTO_ variants (01, 0b) more difficult than the NI(LT)TTO variants. Respondents also found the visual variant (Mode b) consistently more difficult than the corresponding tabulated variant (Mode a). Both these findings are in line with the assessment questions (in Table 2). Participants in DCE_TTO_ variants reported more often to be made uncomfortable by the survey. For the other themes, there were no clear patterns by variant.

## 4 DISCUSSION

This study is the first to experiment a full‐scale health state valuation using an innovative valuation method that is a cross between iterative (LT)TTO and DCE_TTO_. NITTO was developed so that the advantages of TTO and DCE are combined and the disadvantages avoided. Compared to TTO without lead time, NI(LT)TTO is not susceptible to iteration bias, uses the same task for states better and worse than being dead, and does not need to make arbitrary transformations of negative values. Compared to iterative LT‐TTO, NI(LT)TTO is not susceptible to iteration bias and does not need to make arbitrary assumptions to address exhaustion of lead time. All of these advantages also apply to DCE_TTO_, but NILTTTO is less cognitively challenging. However, the presentation of each individual NILTTTO task is more complex compared to a DCE_TTO_ task, because of the lead time.

The study used a single‐stage design and a two‐stage design for NILTTTO. And finally, this study compared two modes of presentation: tabulated, and with a visual aid.

The overall results show that NI(LT)TTO is feasible. Ngene can be used to design NI(LT)TTO surveys; fewer respondents found the tasks difficult compared to DCE_TTO_; the great majority “pass” the logical consistency test; and data can be modelled to produce interpretable coefficients. However, a closer look suggests a few issues for discussion.

A major concern is that the modelled coefficients for the NI(LT)TTO variants predict very low values for the milder states. A negative value for state 22222 (2b, Figure 1, full sample) lacks face validity. It appears that at least some respondents are choosing on the basis of the health states alone, without accounting for the durations. This is in contrast to iterative TTO, where some respondents resist trading off any time in full health for relatively mild states (Robinson, Dolan, & Williams, 1997). In other words, relative to iterative TTO, the way NI(LT)TTO was operationalised in this study seems to draw the respondent's attention away from the sacrifice in duration in full health. The exclusion of non‐traders increases the values, but only by 0.14 on average.

Furthermore, there seems to be no simple pattern across the modes. The predicted values for the select states in Figure 2 (both panels) suggest that there may be complex interactions between the mode, the type, and the state. For example, the two milder states tend to have higher values using Mode (a) with the tabular presentation (but not Type 3, state 33333), whereas the two more severe states tend to have lower values with this mode (but not Type 0). In addition, the differences between 22222 and 55555 (Table 3) are larger using Mode (b) with visual aids for the NITTO variants, but not for DCE_TTO_. In terms of respondents' feedback, the tabular Mode (a) was found clear by significantly more respondents in Types 2 and 3 (Table 2).

However, it should also be noted that Type 0a, which is identical to the DCE_TTO_ used in online studies elsewhere, has also resulted in lower predicted values for mild states. Therefore, the lower values for mild states cannot be attributed entirely to NI(LT)TTO. This raises questions for the reliability of online DCE_TTO_, which is beyond the scope of the current study.

A related point is the high proportion of nontraders. Always choosing either A or B throughout NI(LT)TTO tasks means they are not trading between quality of life and survival, and the exercise generates little information from these respondents. The proportion of respondents who always choose B throughout ranges from 7% (2b) to 23% (1b), suggesting a Type × Mode interaction (Table 2). The lower percentage in variant 2b may suggest this variant is relatively immune from nontrading. Indeed, Figure 3 shows that the latent class with the lowest values has the lowest share (dotted lines) in variant 2b.

On the other hand, the predicted values for state 55555 are not particularly low compared to other studies. This leads to a narrower range between the predicted values of 22222 and 55555 (0.48 for variant 2b; Table 3). Analyses of NI(LT)TTO excluding nontraders do not improve the outcomes (e.g., a range of 0.51 for variant 2b; Table 3), suggesting that the phenomenon is not down to a minority of easily identifiable individuals.

The analysis of early/middle/late tasks in Figure 2 suggests that the responses to NI(LT)TTO tasks are stable (at the aggregate) as respondents work their way through the 12 tasks. On the other hand, DCE_TTO_ data appear to deteriorate through the stages, which disagrees with Mulhern et al. (2014). This may also question feedback from respondents: similar proportions of respondents (around 85%) reported that they could answer a few more tasks, with no indication that the Type 0 respondents may be more fatigued than the rest.

The results of the latent class analysis suggest that there is heterogeneity in the data. Assuming the randomisation of respondents across survey variants was successful, it is natural to assume that the level of heterogeneity in underlying individual preferences is similar across the variants. If so, not all the variation across the variants in Figure 3 should be interpreted to represent heterogeneity in individual preferences. One interpretation is to attribute at least some of it to a heterogeneity in the ability of respondents to deal with different NI(LT)TTO tasks. Another possibility is that the NI(LT)TTO variants (especially Types 1 and 2) are better at capturing preference heterogeneity than Type 0 (DCE_TTO_). Either way, judging on the basis of the difference between predicted values for 22222 and 55555 for the class with the highest share (Class 1; solid line), variants 1a (1.23), 1b (1.08), and 3a (1.55) appear similar to DCE_TTO_ (1.59 for 0a; 1.15 for 0b).

The qualitative data reinforce these findings. First, NI(LT)TTO respondents are more likely to choose full health over nonfull health, even when the duration in full health is very short and the nonfull health state is relatively mild. And, second, respondents find NI(LT)TTO easier than DCE_TTO_. However, the qualitative analysis has limitations. The comments were coded by one person (MK), and there was no formal secondary coding. In addition, content analysis was performed and the comments were counted, which assumes that each comment has the same strength.

To conclude, NI(LT)TTO aims to overcome methodological challenges of iterative (LT)TTO and DCE_TTO_. The data indicate that NI(LT)TTO is easier than DCE_TTO_, generate more stable data, and involve less respondent fatigue. However, in its current forms, it clearly has its own challenges. A particular issue is the effect of visual aids used: the respondents in the variant with visual aids found the choice tasks more difficult than the respondents in the variant without visual aids. This seems to suggest substantial scope for improvement in the way the NI(LT)TTO tasks were presented. Further research is needed to better understand the potential interactions across the mode of presentation, the method (or type) of valuation exercise, and the health state being valued in non‐iterative tasks, especially when conducted online.

## CONFLICT OF INTEREST

The authors have no conflict of interest.

## APPENDIX A ADDITIONAL TABLES

### A.1

**Table A1:** Response rate by variant

	0a	0b	1a	1b	2a	2b	3a	3b
*n* accessing	834	712	1249	1429	1183	1294	1326	1638
*n* excluded due to block not available^a^	0	0	0	168	0	0	267	302
*n* starting but not completing survey	216	119	348	361	282	392	156	436
*n* included in analysis	618	593	901	900	901	902	903	900
Response rate^b^ (%)	74.1	83.3	72.1	71.4	76.2	69.7	85.3	67.4
Time taken for the whole survey (median in seconds)	622	655	521	521	467	585	468	590

a A number of people accessing the survey had to be turned away because the initial set up only allowed up to 1,000 attempts per variant, at which point, survey blocks “ran out.” Subsequently, this was corrected to continue accepting respondents and allocating to blocks.

b Completion rate = *n* included/(*n* accessing – *n* excluded due to block not available)

**Table A2:** Background characteristics and feedback by variant

		0a	0b	1a	1b	2a	2b	3a	3b
Number of respondents		618	593	901	900	901	902	903	900
Age (average; years)		46.6	47.2	46.3	46.2	46.2	45.7	46.8	46.5
Females (%)		53.4	49.1	50.2	50.9	50.7	51.2	51.4	50.9
Employed (%)		53.4	51.8	57.3	57.9	56.7	54.6	54.7	54.6
Degree (%)		51.8	52.6	53.3	54.9	49.6	51.7	51.1	53.6
Own health	Illness (%)	26.4	34.7	27.2	28.0	27.6	29.2	31.1	30.6
	M level 1 (%)	76.1	69.0	76.9	76.3	74.8	74.2	73.4	74.4
	SC level 1 (%)	88.5	85.7	88.5	89.6	89.2	88.4	87.5	88.6
	UA level 1 (%)	75.2	68.5	73.4	74.2	73.9	71.6	72.7	72.6
	PD level 1 (%)	51.6	44.4	52.1	50.3	49.5	49.0	47.3	51.4
	AD level 1 (%)	62.1	56.8	54.2	58.7	56.8	53.0	54.0	57.0
	11111 (%)	36.7	29.7	32.1	33.9	31.7	29.8	30.1	33.4
	11121 (%)	10.8	9.3	9.5	10.0	11.0	10.2	10.1	10.4
	11112 (%)	7.1	8.3	10.0	8.8	10.1	9.8	8.6	8.7
Believe life after death (%)^a^		41.8	47.7	45.6	43.6	42.5	45.7	42.5	44.6

a Percentage replying “Yes, definitely” or “Yes, probably” to the question: “Do you believe in life after death?”

**Table A3:** Baseline model regression results by variant

Variable	0a	0b	1a	1b	2a	2b	3a	3b
MO2 × duration	−0.023	0.002	−0.033***	−0.045***	−0.038***	−0.053***	−0.035***	−0.044***
MO3 × duration	−0.043**	−0.019	−0.037***	−0.052***	−0.031***	−0.056***	−0.039***	−0.052***
MO4 × duration	−0.105***	−0.100***	−0.087***	−0.074***	−0.065***	−0.063***	−0.084***	−0.081***
MO5 × duration	−0.133***	−0.118***	−0.088***	−0.101***	−0.075***	−0.071***	−0.097***	−0.090***
SC2 × duration	−0.002	−0.037**	−0.008	−0.035***	−0.020**	−0.040***	−0.035***	−0.044***
SC3 × duration	−0.039**	−0.050***	−0.012	−0.043***	−0.040***	−0.046***	−0.054***	−0.036***
SC4 × duration	−0.101***	−0.101***	−0.076***	−0.083***	−0.070***	−0.061***	−0.092***	−0.081***
SC5 × duration	−0.130***	−0.142***	−0.087***	−0.102***	−0.082***	−0.070***	−0.103***	−0.081***
UA2 × duration	−0.016	−0.035**	−0.020**	−0.044***	−0.023***	−0.031***	−0.038***	−0.063***
UA3 × duration	−0.044***	−0.034**	−0.006	−0.035***	−0.027***	−0.022**	−0.060***	−0.055***
UA4 × duration	−0.065***	−0.089***	−0.053***	−0.067***	−0.061***	−0.037***	−0.080***	−0.085***
UA5 × duration	−0.137***	−0.128***	−0.047***	−0.061***	−0.045***	−0.038***	−0.075***	−0.079***
PD2 × duration	−0.052***	−0.013	−0.039***	−0.041***	−0.049***	−0.063***	−0.024***	−0.038***
PD3 × duration	−0.083***	−0.037**	−0.047***	−0.056***	−0.055***	−0.052***	−0.055***	−0.034***
PD4 × duration	−0.174***	−0.118***	−0.103***	−0.099***	−0.097***	−0.078***	−0.095***	−0.090***
PD5 × duration	−0.212***	−0.158***	−0.123***	−0.121***	−0.099***	−0.090***	−0.107***	−0.077***
AD2 × duration	−0.039**	−0.063***	−0.033***	−0.041***	−0.043***	−0.050***	−0.029***	−0.051***
AD3 × duration	−0.018	−0.050***	−0.032***	−0.045***	−0.044***	−0.049***	−0.052***	−0.051***
AD4 × duration	−0.131***	−0.118***	−0.084***	−0.099***	−0.078***	−0.085***	−0.096***	−0.089***
AD5 × duration	−0.152***	−0.165***	−0.095***	−0.083***	−0.088***	−0.079***	−0.101***	−0.096***
Duration	0.463***	0.393***	0.227***	0.262***	0.222***	0.230***	0.284***	0.272***
observations (*n*)	14832	14232	21624	21600	21624	21648	21672	21600
Log‐likelihood	−4404.8	−4369.07	−5980.1	−5700.98	−5522.19	−5703.08	−6695.38	−6604.76
Rho‐squared	0.143	0.114	0.202	0.238	0.263	0.24	0.109	0.118
AIC	8851.59	8780.141	12002.19	11443.95	11086.37	11448.17	13432.76	13251.53
BIC	9011.285	8938.969	12169.8	11611.54	11253.98	11615.8	13600.42	13419.11

* *p* < .05.

** *p* < .01.

*** *p* < .001.

**Table A4:** The effect of removing non‐trading respondents

	0a	0b	1a	1b	2a	2b	3a	3b
Number of observations	14712	14064	17856	16176	18216	20064	16392	18024
Duration not *p* < .05	0	0	0	0	0	0	0	0
Interaction not *p* < .05 (*n* out of 20)	3	3	4	0	0	0	0	0
Wrong sign (*n* out of 20)	0	0	0	0	0	0	0	0
Not ordered (*n* out of 20)	1	2	3	3	2	4	1	6
Two worst attributes at L5^a^	PD/AD	AD/PD	PD/AD	PD/SC	PD/AD	PD/AD	PD/AD	AD/M
Two least bad attributes at L5^b^	SC/M	M/UA	UA/M	U/AD	UA/M	UA/M	UA/M	UA/PD
Predicted values $\hat{v}$(22222)– $\hat{v}$(55555)	1.36	1.43	1.35	0.97	0.94	0.51	1.10	0.65

a Worst/second worst dimensions amongst the Level 5 coefficients.

b Least/second least bad dimensions amongst the Level 5 coefficients.
